# A cost effective real-time PCR for the detection of adenovirus from viral swabs

**DOI:** 10.1186/1743-422X-10-184

**Published:** 2013-06-07

**Authors:** Turkiya Al-Siyabi, Khalifa Binkhamis, Melanie Wilcox, Sallene Wong, Kanti Pabbaraju, Raymond Tellier, Todd F Hatchette, Jason J LeBlanc

**Affiliations:** 1 Division of Microbiology, Department of Pathology and Laboratory Medicine, Capital District Health Authority Room 404B Mackenzie Building, 5788 University Ave., Halifax, NS B3H 1V8; 2 Dalhousie University, Halifax, Nova Scotia, Canada; 3 Provincial Laboratory for Public Health, Calgary, Alberta, Canada; 4 University of Calgary, Calgary, Alberta, Canada

**Keywords:** Homogenization, Extraction, Real-time PCR, Adenovirus, Cost analysis

## Abstract

Compared to traditional testing strategies, nucleic acid amplification tests such as real-time PCR offer many advantages for the detection of human adenoviruses. However, commercial assays are expensive and cost prohibitive for many clinical laboratories. To overcome fiscal challenges, a cost effective strategy was developed using a combination of homogenization and heat treatment with an “in-house” real-time PCR. In 196 swabs submitted for adenovirus detection, this crude extraction method showed performance characteristics equivalent to viral DNA obtained from a commercial nucleic acid extraction. In addition, the in-house real-time PCR outperformed traditional testing strategies using virus culture, with sensitivities of 100% and 69.2%, respectively. Overall, the combination of homogenization and heat treatment with a sensitive in-house real-time PCR provides accurate results at a cost comparable to viral culture.

## Introduction

Human adenoviruses (HAdV) are ubiquitous DNA viruses that cause a wide spectrum of illness [1]. The majority of HAdVs cause mild and self-limiting respiratory tract infections, gastroenteritis or conjunctivitis; however, more severe disease can occur such as kerato-conjunctivitis, pneumonitis, and disseminated disease in the immunodeficient host [2-5]. HAdV is increasingly being recognized as a significant viral pathogen, particularly in immunocompromized patients where accurate and timely diagnosis can play an integral part of management [6-12].

HAdV diagnosis can be achieved using virus culture, antigen-based methods (immunofluorescence, enzyme immunoassays or immunochromatography), or nucleic acid amplification tests (NAATs). For respiratory viruses, NAATs are well established as the most sensitive methods for detection and have become front-line diagnostic procedures [7,13-18]. Most commercially available NAATs are highly multiplexed assays and enable simultaneous detection of several respiratory pathogens; however, their poor performance for detecting HAdV emphasizes the need for single target detection [15,17,19]. Adenovirus-specific NAATs have been challenged by the diversity of HAdV species, which now include more than 60 different types [20,21]. Commercial qualitative and quantitative NAATs are available for the detection of all HAdV species and most types, yet these are cost prohibitive for many laboratories. “In-house” real-time PCR assays are relatively inexpensive alternatives to commercial NAATs that provide rapid and accurate results [7,17,18,21-26]. Wong and collaborators [18] developed an in-house real-time PCR assay that has been designed for the detection of all HAdV species. It has been extensively validated using a variety of clinical specimens [17,18].

In addition to the PCR reaction itself, extraction of nucleic acids prior to PCR is also a substantial contributor to cost. Recently, a crude mechanical lysis using silica glass beads (i.e. homogenization) and heat treatment was shown to recover herpes simplex virus DNA from swabs submitted in universal transport media (UTM) [27,28]. While defying the traditional paradigm of specimen processing for molecular testing, homogenization with heat treatment was shown to be a cost effective alternative to nucleic acid extraction. This study evaluated whether the combination of homogenization and heat treatment with an in-house real-time PCR would be a cost effective strategy for the detection of HAdV from viral swabs transported in UTM.

## Materials and methods

### Clinical specimens

In patients suspected of respiratory or conjunctivitis, flocked nasopharyngeal or ocular swabs, respectively, were submitted for adenovirus detection. Swabs were collected by clinicians at the Capital Health District Authority (CDHA) and were submitted to the CDHA microbiology laboratory (Halifax, NS, Canada) between April 2010 and March 2012. The swabs were transported in 3 ml of UTM (Copan Diagnostics Inc., Murrieta, CA) and kept at 4°C for no more than 24 hours prior to processing. Viral cultures were performed as part of routine diagnostic testing by experienced technologists. Following virus culture, specimens were transferred in aliquots into cryotubes (without any identifiable patient information) and the anonymized specimen tubes were archived at −80°C for retrospective molecular analyses. Twenty-seven virus culture-positive specimens and 169 virus culture-negative specimens were randomly selected and tested for the presence of HAdV using a well established in-house real-time PCR assay [18] following recovery of viral DNA was recovered by homogenization with heat treatment or automated nucleic acid extraction.

### Ethical considerations

The World Medical Association (WMA) Declaration of Helsinki is a statement of ethical principles for medical research involving human subjects, including research on identifiable human material and data. Since the purpose of this clinical validation was quality improvement of the laboratory detection of adenovirus and relied exclusively on anonymous human biological materials that did not use or generate identifiable patient information, research ethics board (REB) review was not required based on Chapter 2, article 2.4 of the Tri-Council Policy Statement: Ethical Conduct for Research Involving Humans (2nd edition).

### Viral culture

Viral cultures were performed as part of routine diagnostic testing by experienced technologists in the CDHA microbiology laboratory (Halifax, NS, Canada). Briefly, 500 μl of specimen was inoculated onto cultured A549 cells (ATCC CCL-185), incubated at 37°C in a 5% CO_2_ atmosphere, and monitored daily for the presence of characteristic cytopathic effect (CPE) [29]. If CPE was observed, cells were fixed with acetone and stained using specific fluorescein isothiocyanate (FITC)-labeled monoclonal antibodies in the D^2^ Ultra DFA reagent kit (Diagnostic Hybrids, Athens, OH). In absence of CPE, cells were fluid changed on day 7 and incubated for an additional 7 days. On day 14, the culture was discontinued and a terminal stain was performed. A549 cells were propagated in Nutrient Mixture F-12 Ham with L-glutamine (Sigma-Aldrich Canada Ltd., Oakville, ON) supplemented with 1% fetal calf serum (Hyclone, Thermo Fisher Scientific, Ottawa, ON), 2 μg/ml amphotericin B (Sigma-Aldrich), 25 μg/ml ampicillin (Novapharm Ltd, Toronto, ON), and 1 mg/ml vancomycin (Sigma-Aldrich).

For quantification, 10-fold dilutions of HAdV-C, type 6 (strain Tonsil 99, ATCC VR-6) were inoculated onto 96-well plates in volumes of 100 μl. Cells were maintained as described above and after 14 days were subjected to direct immunofluorescence (DFA) to determine the 50% tissue culture infective dose (TCID_50_). Results were expressed as TCID_50_/ml and represent 8 replicates obtained in four independent experiments (n = 32).

### Homogenization with heat treatment

Prior to molecular testing, viral DNA was recovered from specimens using either homogenization with heat treatment as previously described [27], or using a commercial nucleic acid extraction as recommended by the manufacturer. For homogenization, 500 μl of specimen and 0.5 g of various sized acid-washed silica beads: ≤106 μm; 150–212 μm; 719–1180 μm at a ratio of 3:2:1 (Sigma-Aldrich, Oakville, ON) were placed on a Fastprep-24 homogenizer (MP BioMedicals, Solon, OH) at 6.5 m/s for 45 s. Following a brief centrifugation at 14,000 × g for 1 min, 200 μl of the supernatant was diluted in two volumes of TE buffer (10 mM Tris–HCl, 1 mM EDTA, pH 8.0). The homogenate was then heated at 95°C for 15 min, cooled to room temperature, and 5 μl was subjected to adenovirus real-time PCR.

### Nucleic acid extraction

Automated extractions were performed on 200 μl of specimen using a MagNA Pure Total Nucleic Acid Isolation kit (Roche Diagnostics, Mannheim, Germany) on a Roche MagNAPure LC instrument. The elution volume was 100 μl. Specimens with discordant results during method comparison were subjected to a manual DNA extraction using a QIAamp DNA Blood Mini kit (Qiagen, Toronto, ON) with a sample volume of 200 μl. The DNA was eluted in 100 μl, and concentrated 10-fold using a Qiagen MinElute PCR purification kit. Plasmid DNA, used for the internal control, was purified from a 5 ml overnight culture using a QIAprep Spin Miniprep kit (Qiagen) as recommended by the manufacturer. For molecular typing, amplicon was purified using a QIAquick Gel Extraction Kit (Qiagen) with a final elution volume of 50 μl. All nucleic acid extractions were performed using manufacturers’ instructions. Nucleic acids were used immediately following extraction and aliquots were placed at −80°C for long-term storage.

### In-house real-time PCR

The real-time PCR has been extensively validated using respiratory specimens [18]. To facilitate workflow in the CDHA microbiology laboratory (Halifax, Nova Scotia, Canada), the in-house assay was optimized for amplification and detection on a Roche LightCycler 2.0 platform. Real-time PCR was performed as duplex reactions with primers and probes (Table 1) targeting the adenovirus hexon gene and an exogenous internal control. For adenovirus, two sets of primers and probes were used to span the genetically diverse adenovirus types [17,18]. Primers were synthesized by Sigma Genosys (Oakville, ON). Probes for adenovirus and the internal control were purchased from BioSearch Technologies (Novato, CA) and TIB MOLBIOL LLC (Adelphia, NJ), respectively.

**Table 1:** Nucleotide sequences of primers and probes used in this study

**Name**	**Sequence (5′ to 3′)**	**Reference**
AdV2F	CCA GGA CGC CTC GGA GTA	[18]
AdV2R	AAA CTT GTT ATT CAG GCT GAA GTA CGT	[18]
AdV2pr	FAM- AGT TTG CCC GCG CCA CCA CCG – BHQ1^*^	[18]
AdV4F	GGA CAG GAC GCT TCG GAG TA	[18]
AdV4R	CTT GTT CCC CAG ACT GAA GTA GGT	[18]
AdV4pr	FAM- CAG TTC GCC CGY GCM ACA G – BHQ1^*^	[18]
FGFP	TGA TAC CCT TGT TAA TAG A	This study
RGFP	ATT GTG TGA GTTATA GTT G	This study
GFPpr1	GGT ATT GAT TTT AAA GAA GAT GG – FAM^**^	This study
GFPpr2	LC705 – CAT TCT TGG GCA CAA ATT GGA- Ph^**^	This study
AD1SEQ	CTG ATG TAC TAC AAC AGC ACT GGC AAC ATG GG	[32]
AD2SEQ	GCG TTG CGG TGG TGG TTA AAT GGG TTT ACG TTG TCC AT	[32]
F14MUT	TCT GCG GGT AAT TTA CTA ACT AG	This study
R14MUT	ATC TCC TGT GTT CCA GGA CCA	This study

^*^ The hydrolysis probes were labeled with 6-carboxyfluorescein (FAM) at the 5’end; however, the 3’end was labeled with a Black Hole Quencher 1 (BHQ1) instead of the carboxytetramethyl-rhodamine (TAMRA) quencher previously described [18].

^**^ Abbreviations for the hybridization probes targeting the internal control (pGFP) are as follows: [FAM], 6-carboxyfluorescein; [LC705], LightCycler-Red 705; Ph, 3′-phosphate.

The internal control, termed pGFP, is added to each reaction to monitor for the presence of PCR inhibitors. pGFP is a pMK-derived plasmid with a fragment of the gene encoding green fluorescence protein (GFP). The construct was synthesized, assembled, and transformed into *Escherichia coli* K12 by Life Technologies (Burlington, ON). The final construct was verified by DNA sequencing and restriction endonuclease digestion. *E. coli* harboring pGFP was inoculated into Luria Bertani broth supplemented with 50 μg/ml kanamycin. Plasmid DNA was purified from a 5 ml overnight culture and plasmid DNA was quantified by spectrophotometry. Ten-fold serial dilutions were used as template for the in-house real-time PCR. An inverse linear relationship (y = −3.3916× + 40.275; R^2^ = 0.9982) was generated by plotting crossing points (Cp) values against plasmid concentration (data not shown). The linear range spanned Cp values ranging from 7 to 37, corresponding to concentrations of 10^0^ to 10^9^ copies per μl, respectively. For each PCR reaction, approximately 2000 copies were added.

Real-time PCR assay was performed using the LightCycler DNA Master HybProbe kit (Roche Diagnostics) in 20 μl reactions consisting of: 5 μl of template, 1 × LightCycler FastStart mix, 3 mM MgCl_2_; 0.5 units of heat-labile uracil-N-glycosylase [30]; 5 μl the internal control at 400 copies/μl; 400 nM of each adenovirus primer (AdV2F, AdV2R, AdV4F, AdV4R) and 200 nM of probe (AdV2pr and AdV4pr); and 500 nM of each pGFP primer (FGFP and RGFP) and 300 nM of each probe (GFPpr1 and GFPpr2) (Table 1). Amplification and detection were performed using the LightCyler 2.0 instrument under the thermocycling conditions described for the Roche HSV-1/2 detection kit: initial activation at 95°C for 10 min, followed by 45 amplification cycles of denaturation at 95°C for 10 s, annealing at 55°C for 15 s, and elongation at 72°C for 15 s. Following amplification, melting temperature (Tm) analysis was performed by measuring the fluorescent signal during the following cycling profile: 95°C for 0 s, 40°C for 60 s, and 80°C for 0 s with a 0.2°C/s transition. Fluorescence was acquired at the annealing stage during amplification and continuously during the melting curve. Cp and Tm values were determined using software provided by the manufacturer. The 530 nm (adenovirus) and 705 nm (pGFP) channels were analyzed for presence or absence of target. PCR inhibition was suspected by either loss of positivity in the 705 nm channel, or a shift in Cp values greater than two standard deviations (Cp ≥ 1.0) from the value obtained with the negative control.

### Commercial real-time PCR

To resolve discrepant results obtained between the in-house PCR assay and virus culture, or quantify the adenovirus DNA during evaluation of the analytical sensitivity, the Adenovirus R-Gene kit (Argene Inc., Sherley, NY) was used according to the manufacturer’s protocol following a manual DNA extraction. This internally controlled quantitative real-time PCR assay targets the hexon gene of adenovirus, and is validated for detection of types 1 to 52 [7]. The kit contains: a ready-to-use premix contains (primers, probe, polymerase, and buffer) needed for amplification, 4 quantification standards (at 50, 500, 5,000, and 50,000 copies/reaction), and a sensitivity-control at 10 copies/reaction. Results were expressed as the number of copies per reaction.

### Analytical specificity, limit of detection, and reproducibility

**Figure 1:**
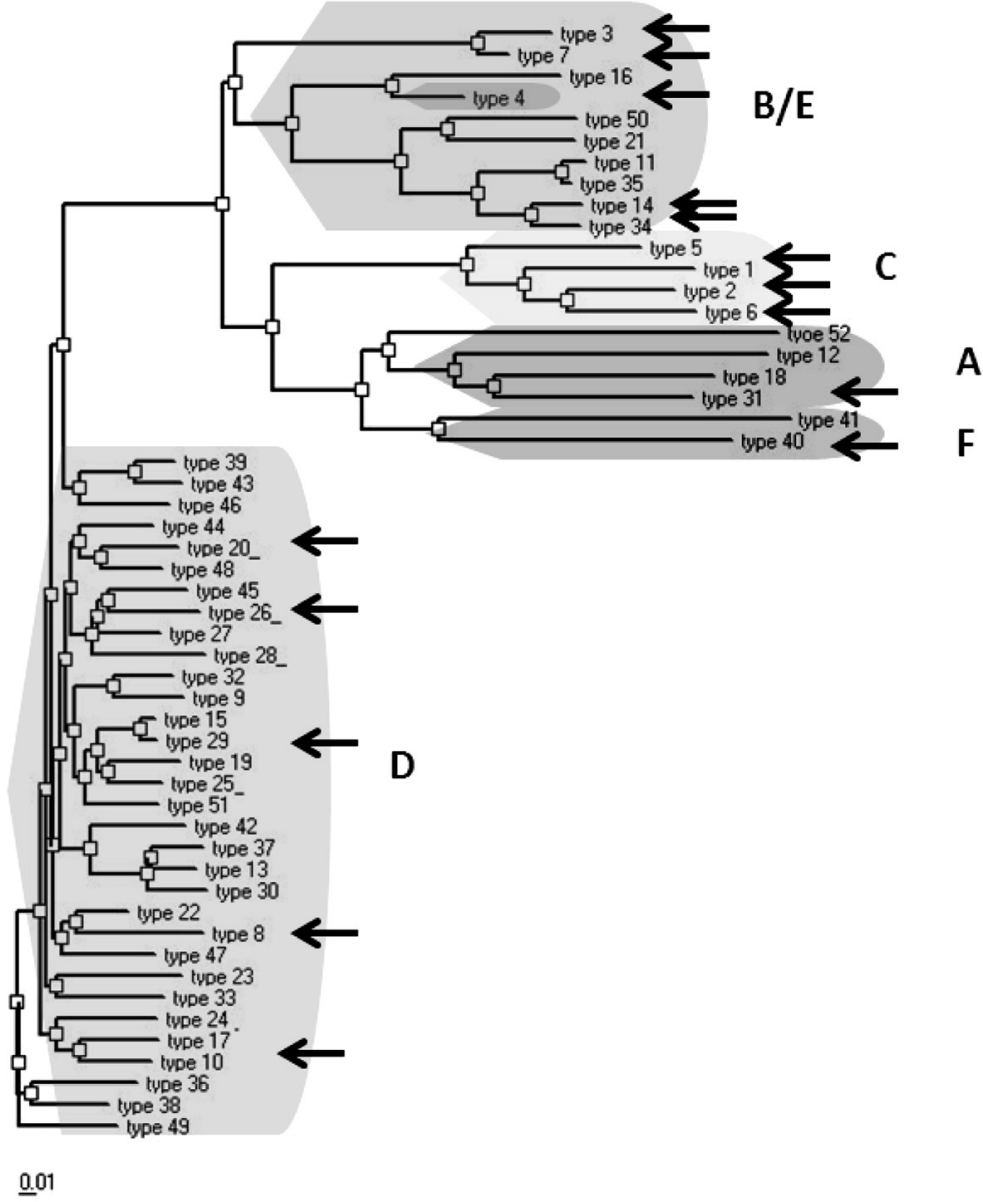
**Phylogenetic tree derived from hexon gene sequences.** The HAdV types used in the specificity panel are indicated by arrows. Clades are shaded to depict species **A** to **F**.

The analytical specificity was first determined *in silico* by performing a Basic Local Alignment Search Tool (BLAST) for primers, probes, and entire amplicon sequences using the National Center for Biotechnology Information website (http://www.ncbi.nlm.nih.gov). In addition, high titer nucleic acids were extracted from a panel of microorganisms chosen based on their ability to cause similar diseases or their potential for being found in the clinical specimen as a pathogen or normal flora (Table 2). To test for assay inclusivity, adenoviruses spanning the various species and types were tested by the in-house real-time PCR: [HAdV-A type 31; HAdV-B types 3, 7, 14, 34; HAdV-C types 1, 2, and 6; HAdV-D (type 8, 10, 20, 26, and 29); HAdV-E type 4, HAdV-F type 40] (Figure 1 and Table 2).

**Table 2:** Organisms used for the specificity panel

**Name**	**Real-time PCR Result**	
	**Nucleic acid extraction**	**Homogenization and heat treatment**
HAdV-A, type 31	+	+
HAdV-B, type 3	+	+
HAdV-B, type 7	+	+
HAdV-B, type 14	+	+
HAdV-B type 34	+	+
HAdV-C, type 1	+	+
HAdV-C, type 2	+	+
HAdV-C, type 6	+	+
HAdV-D, type 8	+	+
HAdV-D, type 10	+	+
HAdV-D, type 20	+	+
HAdV-D, type 26	+	+
HAdV-D, type 29	+	+
HAdV-E, type 4	+	+
HAdV-F, type 40	+	+
Influenza A (pandemic H1N1, seasonal H1N1 and seasonal H3N2)	-	-
Influenza B (Victoria and Yamagata lineages)	-	-
Parainfluenza virus (types 1 to 4)	-	-
Respiratory syncytial virus (types A and B)	-	-
Human coronaviruses (229E, OC43, NL63, and HKU1)	-	-
Human metapneumovirus	-	-
Rhinovirus (A, B or C)	-	-
Human bocavirus	-	-
Human herpesvirus (types 1–5, 6a, 6b, 7 and 8)	-	-
*Streptococcus pneumoniae*	-	-
*Haemophilus influenzae*	-	-
*Moraxella catarrhalis*	-	-
*Legionella pneumophila*	-	-
*Mycoplasma pneumoniae*	-	-
*Staphylococcus aureus*	-	-
*Staphylococcus epidermidis*	-	-
*Pseudomonas aeruginosa*	-	-
*Escherichia coli*	-	-
*Neisseria gonorrhoeae*	-	-
*Chlamydia trachomatis*	-	-

*Virus and bacterial strains were obtained from the microbiology laboratory at CDHA (Halifax, NS) or the Provincial Laboratory for Public Health (Calgary, Alberta).

The analytical sensitivity (or limit of detection, LoD) of the homogenization with heat treatment or nucleic acid extraction, in combination with the real-time PCR, was determined using 10-fold serial dilutions (in UTM) of a cultured HAdV-C type 6. Each dilution was simultaneously processed by both extraction methods, and an aliquot immediately inoculated onto A549 cells for virus culture. The LoD was defined by Probit analysis [31] using triplicate values obtained in four independent experiments by two different operators (n = 24). Each virus dilution was expressed as TCID_50_/ml in the original sample. The virus dilutions were also quantified using a commercial real-time PCR and expressed as target copies/reaction for each assay. Intra- and inter-assay reproducibility were calculated for each dilution and expressed as % coefficients of variation (%CV).

### Method comparison

The performance of each method was compared to a modified gold standard to determine sensitivity, specificity, accuracy and precision. A case was defined by concordant results (positive or negative) between at least two assays. To resolve discrepant results obtained between the in-house real-time PCR assay and virus culture, DNA was extracted manually and was subjected to commercial real-time PCR.

### Molecular typing of positives specimens

The 27 virus culture-positive specimens were subjected to PCR targeting the conserved segments surrounding the hypervariable region 7 (HVR7) of the hexon gene [32]. PCR reactions were performed in 25 μl volumes using illustra Hot Start Ready-to-go (RTG) beads (GE Healthcare UK Ltd., Bukinghamshire, UK), supplemented with 1 μM of each primer (AD1SEQ and AD2SEQ; Table 1), and an additional 0.5 mM MgCl_2_. Thermocycling conditions were as follows: initial activation at 95°C for 10 min; 40 cycles of denaturation at 95°C for 1 min; annealing at 50°C for 1 min; extension at 72°C for 1 min; and a final extension of 5 min at 72°C. Amplifications were performed on a DNA engine dyad thermocycler (Bio-Rad Laboratories Ltd., Mississauga, Ontario, Canada) and amplicons were resolved by 1.5% agarose gel electrophoresis with ethidium bromide staining. Amplicons corresponding to the expected size (approximately 600 bp) were excised using a sterile scalpel and were purified using a QIAquick Gel Extraction Kit (Qiagen). Purified amplicon DNA was submitted for DNA sequencing at the York University Core Molecular Biology and DNA Sequencing Facility (Toronto, Ontario, Canada). Sequencing was performed using BigDye Terminator chemistry on the Applied Biosystems 3130 × L DNA Sequencer. Type designation was undertaken by BLAST analysis, and confirmed by comparison to a database generated from sequences obtained from GenBank [32]. Sequence analysis and multiple sequence alignments (ClustalW analysis) were performed using the Seqman and Megalign components of Lasergene 6 software (DNASTAR, Madison, WI). The phylogenetic tree was inferred using a neighbor-joining (NJ) method with bootstrapping analysis for n = 1000.

### Statistical analysis

Chi-square and two-tailed Fisher’s exact tests were used to compare proportions in 2-by-2 contingency tables. Confidence intervals (99%) for the estimated parameters are computed by a general method based on “constant chi-square boundaries” [33]. Agreement between assays was measured using kappa statistics. The Statistical Package for Social Sciences (SPSS) software v.10 was used and *P* ≤ 0.01 was used to denote a statistically significance.

## Results

### Analytical specificity, limit of detection, and reproducibility

BLAST searches of primers and probes targeting the adenovirus hexon gene the internal control sequences revealed that these were highly specific targets. In fact, no cross reactions were observed with high-titer nucleic acids extracted from other respiratory viruses or bacteria (Table 2). The in-house real-time assay was able to detect serogroups A to F, including a variety of genetically diverse types: 1, 2, 3, 4, 6, 7, 10, 20, 26, 31, and 40 (Figure 1 and Table 2).

**Figure 2:**
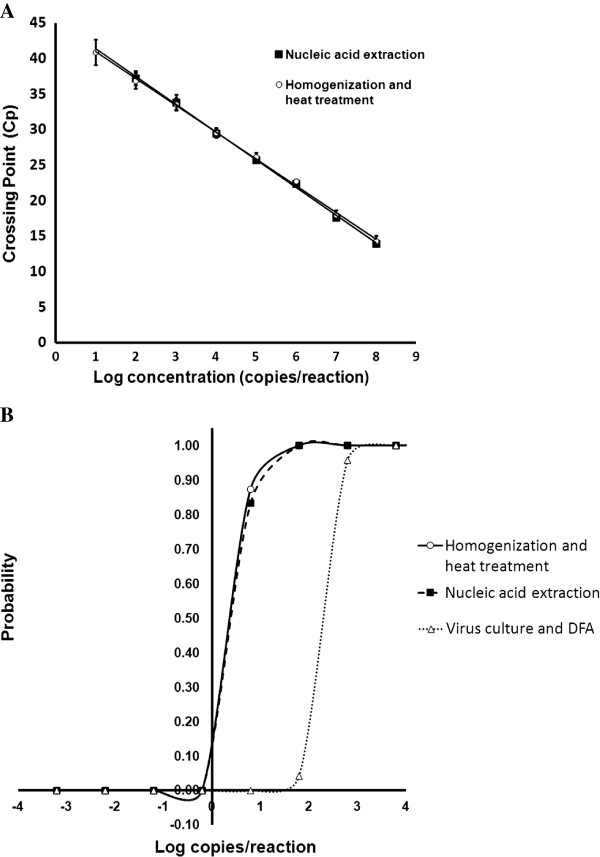
**Analytical sensitivity of the in-house real-time PCR.** Prior to amplification, 10-fold serial dilutions of HAdV-C type 6 were processed by homogenization and heat treatment (open circles, solid line), or nucleic acid extraction (filled squares, dashed line). In both cases, equivalent results were obtained in respect to: **A**) the linear range; and **B**) the LoD determined by Probit analysis (n = 24). At a probability of 95%, the LoD for the homogenization- and nucleic acid extraction-based protocols were 12 copies/reaction (log_10_ = 1.08) and 18 copies/reaction (log_10_ = 1.08), respectively. The same dilutions used for inoculate virus culture and DFA staining (indicated by open triangles, dotted line) were also quantified and demonstrated a LoD of approximately 380 copies/ml (log_10_ = 2.58).

As seen in Figure 2, the performance of the in-house PCR following the homogenization- or nucleic acid extraction-based protocols was equivalent. For each method, overlapping linear relationships were observed (y = −3.7668 × + 44.733; R^2^ = 0.9987 compared to y = −3.9058 + 45.313; R^2^ = 0.9985, respectively) that spanned eight orders of magnitude with Cp values ranging from 14 to 40 (Figure 2A). The intra- and inter-assay reproducibility of the real-time PCR following homogenization and heat treatment ranged from 0.03 to 4.80%, and 1.45 to 3.79%, respectively. Similarly, intra- and inter-assay reproducibility of following the nucleic acid extraction protocol ranged from 0.2 to 2.15% and 0.85 to 3.15%. As expected, the highest %CV values observed for both methods were with virus dilutions near the LoD.

For HAdV-C type 6, the LoD for virus culture was 0.2 TCID_50_/ml. The in-house real-time PCR was reproducibly positive following nucleic acid extraction or homogenization with viral stock dilutions corresponding to 0.02 TCID_50_/ml (24/24 and 24/24, respectively), and positive PCR reactions were frequently observed using virus dilutions of 0.002 TCID_50_/ml (20/24 and 21/24, respectively). Virus stock dilutions were quantified using commercial real-time PCR assay, and the LoD for homogenization or nucleic acid extraction-based protocols were shown to be approximately equivalent (Figure 2). With a probability of 95%, the LoD for the homogenization- and nucleic acid extraction-based protocols were 12 copies/reaction (log_10_ = 1.08) and 18 copies/reaction (log_10_ = 1.08), respectively (Figure 2B). Dilutions corresponding to the LoD for virus culture were also quantified by real-time PCR and estimated at approximately 380 copies/reaction (Figure 2B).

### Method comparison using clinical specimens

**Figure 3:**
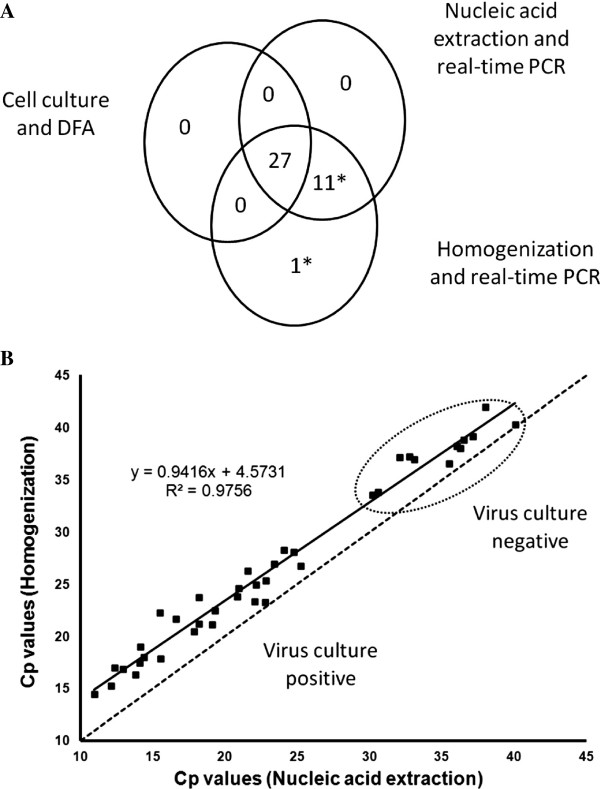
**Comparison of the positive results obtained with virus culture and the two molecular assays. A**) In each field of the Venn diagram, the number of positive specimens is given. *All virus culture-negative/PCR-positive specimens were resolved as true positives by a commercial DNA extraction and real-time PCR. **B**) The Cp values obtained by real-time PCR in 27 adenovirus-positive specimens following homogenization- or nucleic acid extraction-based protocols displayed a correlation of 97.6% (solid line). The dashed line represents a correlation of 100%. The dotted oval highlights Cp values obtained with specimens that were PCR-positive but culture-negative.

Of the 196 clinical specimens, 157 concordant negative and 27 concordant positive results were obtained when comparing virus culture to the in-house PCR following either of the two extraction methods (Figure 3A and Table 3). Real-time PCR generated 12 additional positive results that were later resolved as true positives using a manual DNA extraction and a commercial real-time PCR (Figure 3A). All 12 PCR-positive culture-negative results were detected following homogenization protocol, whereas 11 were detected following nucleic acid extraction (Figure 3A). The single discordant result between the molecular assays had a Cp value of 37.22, suggesting that it may be attributed to sampling error (Poisson distribution) at low concentrations of template [34]. Since the internal control also failed to amplify in this sample, the negative result could also be attributed to PCR inhibition. Upon repeat processing by automated and manual nucleic acid extractions, positive results were obtained. Therefore, the original specimen result was considered a false negative. Overall, compared to the modified gold standard, the sensitivity of the in-house real-time PCR following homogenization with heat treatment or nucleic acid extraction was approximately equivalent at 100% (89.7–100%) and 97.4% (86.4–97.4%), respectively (Table 3). In contrast, the sensitivity of virus culture was only 69.7% (56.0–69.2%) (Table 3). The accuracy of each method was 100% (95.6–100%), 99.5% (95.1–99.5%), and 93.9% (88.6–93.9%), respectively (Table 3). All assays showed a high degree of specificity and precision (Table 3).

**Table 3:** Summary of the method performance characteristics compared to the modified gold standard^*^

**Method**	**Sensitivity (%; 99% CI)**	**Specificity (%; 99% CI)**	**Accuracy (%; 99% CI)**	**Precision (%; 99% CI)**	**Kappa (99% CI)**
Virus culture	69.2 (56.0-69.2)	100 (96.7-100)	93.9 (88.6-93.9)	100 (80.8-100)	0.783 (0.596-0.783)
Nucleic acid extraction and real-time PCR	97.4 (86.4-97.4)	100 (97.3-100)	99.5 (95.1-99.5)	100 (88.6-100)	0.984 (0.844-0.984)
Homogenization with heat treatment and real-time PCR	100 (89.7-100)	100 (97.4-100)	100 (95.6-100)	100 (89.7-100)	1.000 (0.871-1.000)

^*^ A case was defined by concordant results (positive or negative) between at least two assays. A manual nucleic acid extraction and commercial real-time PCR was used to resolve discrepant results. *TP* true positive, *TN* true negative, *FP* false positive, *FN* false negative, *CI* confidence interval.

When comparing Cp values for the positive results obtained with the real-time PCR following both extraction methods, a linear relationship was observed (y = 0.9416 × + 4.5731; R^2^ = 0.9756) (Figure 3B). Cp values for homogenization with heat treatment were consistently higher than those obtained using the nucleic acid extraction; however, no significant differences in sensitivity (analytical or clinical) were observed (Figure 2 and Table 3). As expected, virus culture-positive specimens had positive PCR results with low Cp values, whereas the virus culture-negative specimens had PCR-positive results with Cp values greater than 30 (Figure 3B).

### Molecular typing of positives specimens

DNA extracted from the 39 real-time PCR positive specimens were subjected to a conventional PCR targeting the conserved segments surrounding the HVR7 of the hexon gene [32]. Successful sequences were obtained from DNA extracted from the 27 specimens that were both virus culture and real-time PCR-positive. A type could be assigned using multiple sequence alignment of sequences derived from GenBank, as previously described [32]. Individual BLAST analysis yielded similar results. Three serogroups were observed: B (types 3, 7, 14, and 34), C (types 2 and 6), and D (types 8, 10, and 29). The predominant types observed were: 3 (37.0%), 29 (18.5%), 2 (14.8%), and 8 (1.1%). The conventional PCR was unable to amplify the target sequences from DNA extracted from the 12 virus culture-negative/real-time PCR-positive specimens. The Cp values for these specimens ranged from 30 to 40, suggesting only low quantities of virus were present (Figure 3).

### Mutation analysis in adenovirus type 14

**Figure 4:**
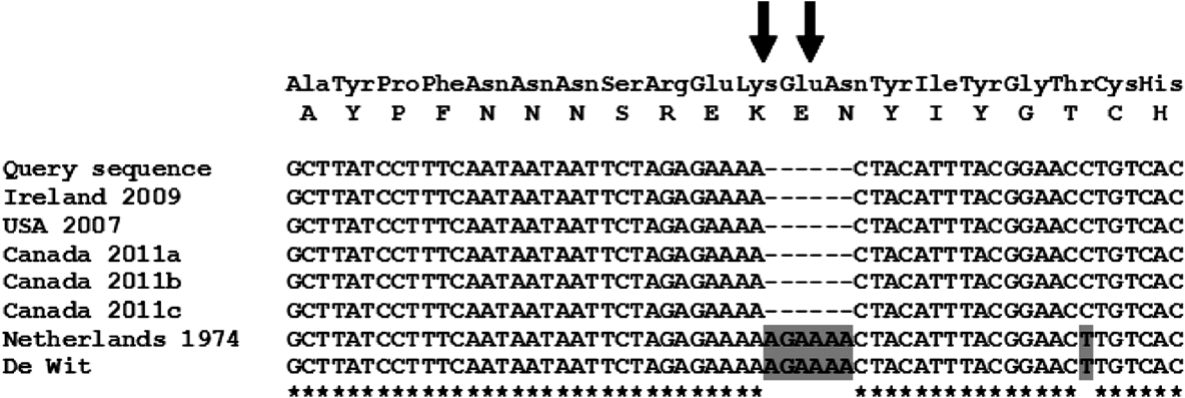
**Human adenovirus fiber knob gene alignment.** Compared to the prototypic type 14p (De Wit) sequence, a 6-bp deletion is clearly visible in all HAdV type 14p1 sequences. Black arrows demarcate the K250-E251 deletion. The query sequence from this study aligns with the HAdV type 14p1 sequences and also contains the 6-pb deletion. GenBank numbers are as follows: Ireland 2009 [Dublin/2009 (HQ163915.1/GI:307147785)]; USA 2007 [Portland/2971/2007 (FJ841909.1 GI:268588942)]; Canada 2001a [Canada/RV1368/2011 (JQ815084.1 GI:395135725)]; Canada 2001b [Canada/RV1360/2011 (JQ815083.1 GI:395135723)]; Canada 2011c [Canada/RV1370/2011 (JQ815085.1 GI:395135727)]; Netherlands 1974 [Netherlands/16845/1974 (FJ841910.1 GI:268588944)]; de Wit strain ATCC VR-1091 (AY803294.1 GI:57115621).

DNA sequencing was also used to distinguish the prototypic HAdV type 14p (strain De Wit) from newly emerged type 14p1. Adenovirus type 14p1 has been associated with severe disease in Europe and the North America [2-5]. While the hexon HVR7 sequences obtained in this study share 100% identity with HAdV type 14p1, only two mutations (G1341A and G1491A) separate types 14p1 from 14p in this region. To further characterize the virus, the fibre knob gene was sequenced with primer pair F14MUT and R14MUT (Table 1), using reaction conditions, thermocycling parameters, and DNA sequencing as described for the molecular typing. Compared to wild-type 14p, the fiber knob gene of HAdV type 14p1 displays a 6-bp deletion (referred to as the K250-E251 deletion) [4,35,36]. The adenovirus type 14 from this study harbored the characteristic 6-bp deletion, consistent with HAdV type 14p1 (Figure 4).

### Monitoring for PCR inhibition

An exogenous internal control was used in this study which is non-competitive (contains a primer pair that does not target adenovirus). The addition of the internal control and primers and probes to the in-house PCR reaction did affect the analytical sensitivity of the assay (data not shown). Since the internal control was added at the level of PCR, both extraction methods could be directly evaluated for the presence of PCR inhibitors. Despite a subsequent heat treatment and dilution step, homogenization is a crude method to recover viral DNA and may not sufficient to remove or inactivate PCR inhibitors. Amplification of the internal control in adenovirus-negative specimens is consistent with a true negative result and not simply attributed to PCR inhibition. PCR inhibition was suspected by either loss of positivity in the 705 nm channel, or a shift in Cp values greater than two standard deviations (which corresponds to approximately ±1.0 Cp) from the value obtained with the negative control. This value was established previously, where the internal control Cp values from 150 consecutive HSV-negative specimens were compared by homogenization and heat treatment or nucleic acid extraction [27]. This cutoff value remains true for the internal control used in this study.

Since the in-house PCR was performed as a duplex with an internal control added at the level of PCR, the 196 clinical specimens processed following homogenization and heat treatment or nucleic acid extraction could be monitored directly for the presence of potential PCR inhibitors. Potential inhibitory substances were observed in two distinct cases: the first was a specimen that had been processed by homogenization with heat treatment, and the second, in a specimen subjected to nucleic acid extraction. In both cases, PCR inhibition was not observed upon repeat processing, suggesting either a processing error had occurred or the PCR inhibitor was labile [37]. Therefore, PCR inhibition could not be proven or excluded. As a result, the rate of possible PCR inhibition with either extraction method was equivalent at 0.51% (1/196).

### Cost analysis

At CDHA (Halifax, NS, Canada), the average number of specimens submitted yearly for adenovirus testing is 312 (range 208 to 466 for years 2009 to 2012) and the turnaround time for virus culture can be up to 14 days. A cost analysis was performed that assumed a more practical approach of bi-weekly molecular testing (3–5 specimens with positive, negative and reagent controls). Excluding labor, the average cost of a commercial PCR following nucleic acid extraction would range from $45 to $55 (CAD) per specimen. In comparison, the in-house real-time PCR following a nucleic acid extraction would reduce the cost approximately ~2-fold ($21.44 to $25.97). Replacement of the nucleic acid extraction with the homogenization-base protocol further reduces the cost ~2-fold ($8.84 to $10.97), which is comparable to the average cost of virus culture ($9.47 to 11.64). The time require for bi-weekly processing for either molecular methods is ~5 h/week, which is far lower than the time required for weekly maintenance and processing of specimens using cell culture and DFA staining.

## Discussion

NAATs like real-time PCR have revolutionized the detection of human pathogens in clinical microbiology laboratories. Rapid specimen throughput and excellent performance characteristics make them an appealing alternative to traditional culture methods; however, cost limits their use in many clinical laboratories. Both the recovery of nucleic acids using extraction and the PCR reaction itself contribute to the cost. We have shown that combining a crude extraction method like homogenization with heat treatment [27] and an in-house real-time PCR [18] is a cost effective strategy for the detection HAdV from swabs submitted in UTM. Homogenization uses multidirectional motion to disrupt cells through contact with silica beads and the heat treatment [27,28]. In combination with a subsequent heat treatment to inactivate heat-labile PCR inhibitors, this crude mechanical lysis had been shown to be a cost-effective method to recover viral DNA from swabs transported in UTM [27]. The performance characteristics of this approach were equivalent to using traditional nucleic acid extraction and both molecular methods far exceeded those obtained with virus culture.

Replacing the nucleic acid extraction with the homogenization protocol did not affect the analytical (or clinical) sensitivity of the real-time PCR (Figure 2 and Table 3). Using dilutions of HAdV-C type 6, the LoD for the homogenization protocol was approximately 12 copies/reaction, was consistent with previously reported values (22–33 copies/reaction) for HAdV types 2 and 4 [18]. This analytical sensitivity is approximately 32-fold more sensitive than the estimated LoD for virus culture. Furthermore, positive results could be even obtained at 6 copies/reaction with a probability of 87.5% (Figure 2B). While no significant differences were observed between the molecular assays, both demonstrated a high level of analytical sensitivity.

When comparing 196 clinical specimens using a modified gold standard, the in-house PCR following homogenization and heat treatment or nucleic acid extraction demonstrated similar sensitivities of 100% and 97.4%, respectively (Table 3). This far surpasses the performance of virus culture at 69.2%. The 30% increase in positivity is consistent with the ~32-fold increase in analytical sensitivity and is not surprising since similar results were observed when transitioning other viruses from culture to NAATs [38-41]. When comparing positive results from the in-house real-time PCR, Cp values obtained following the homogenization protocol were consistently higher than those obtained following nucleic acid extraction (Figure 3B). However, the analytical and clinical sensitivities of each assay were not significantly different (Figure 2 and Table 3). It should be noted that all virus culture-negative/PCR-positive specimens had Cp values greater than 30, corresponding to viral loads that fell below the LoD for virus culture (Figure 3B).

The homogenization- or nucleic acid extraction-based protocols both showed excellent analytical specificity, with no cross-reactions from other organisms (Table 2). Both methods were able to detect diverse HAdV types spanning all the different species (Figure 1 and Table 2). Of the virus culture-positive specimens, the most predominant types detected were 3, 29 and 2, belonging to species B, D and C, respectively. These HAdV types are well-recognized causes of acute respiratory tract and ocular infections and are consistent with the distribution reported by others regions in Canada [42,43]. Interestingly, a variant of HAdV type 14, termed 14p1, has been described as an emerging pathogen associated with outbreaks and sporadic cases of acute respiratory disease in Europe and the United States [2-5]. While most recorded cases were mild infections, severe disease and deaths have occurred. HAdV type 14p1 has a characteristic 6-bp deletion (K250-E251) in the fiber knob gene [4,35,36]. The adenovirus type 14 from this study was consistent with type 14p1 and harbored these mutations (Figure 4). While there has been a number of reports of type 14p1 circulating in the US and Europe, this variant has only once been reported in Canada [4]. The first adenovirus 14p1 cases in Canada were reported from Nova Scotia’s neighboring province, New Brunswick, and included one fatality (Figure 4) [4]. The specimen identified as 14p1 in this study was obtained from a fatal case dating back to same time period as the New Brunswick cases. Further epidemiological investigations are underway. While severe and fatal cases associated with type 14p1 have been reported, similar outcomes have been reported with many other common HAdV types [6,7,10,44]. The most likely culprit of disease severity is the immune status of the host, not the adenovirus type or species.

It should be noted that the thermocycling conditions for the adenovirus PCR were modified to allow simultaneous processing of other real-time PCR assays (HSV and VZV) in the CDHA microbiology laboratory [18]. Simultaneously processing of multiple PCR assays on the same LightCycler instrument allows more efficient batch testing when equipment availability is limited. Interestingly, these modifications allowed the detection of HAdV type 31 which had previously been problematic on an ABI instrument [18]. Difference between assays can be attributed to a numerous factors (i.e. instrumentation, kits, etc.); however, the most likely explanation in this case is the annealing temperature. Using the original PCR protocol [18], HAdV type 31 could only be detected when the annealing temperature was reduced from 60°C to 57°C [18]. The annealing temperature in this study is 55°C. Using conditions described in this study, the detection of HAdV type 31 has now been replicated in both collaborating laboratories.

A limitation of this study is that the validation of homogenization was only performed using swabs in UTM. Future experiments will need to examine whether homogenization can be applied to other relevant specimen types (urine, stool, blood and tissue); however, the real-time PCR following a nucleic acid extraction has been shown to be effective for this purpose [18,21]. Secondly, the performance characteristics of homogenization may vary between PCR assays and should not be implemented without proper validation [27]. While homogenization with heat treatment has shown to be effective for the recovery of viral DNA from HAdV (this study), HSV [27], and varicella zoster virus, decreased sensitivity was observed for enveloped RNA viruses like mumps and influenza viruses ([24,45] LeBlanc, J. unpublished data).

Homogenization and heat treatment showed performance characteristics equivalent to a commercial nucleic acid extraction for the detection of HAdVs. In combination with a sensitive in-house real-time PCR, homogenization with heat treatment generated results far superior than virus culture, and at a comparable cost. By modifying the thermocycling conditions to those used by other assays in the CDHA microbiology laboratory, it further streamlined workflow and facilitated transition from virus culture to molecular testing. Compared to virus isolation and propagation using culture, molecular testing also further reduces the risk of laboratory-acquired infections [46]. Overall, homogenization with heat treatment combined with a sensitive in-house real-time PCR is a cost-effective method for the detection of HAdVs.

## Competing interests

The authors declare that they have no competing interests.

## Authors’ contributions

JL conceived the study. JL, TH and RT participated in its design and coordination. TA, KB, and JL carried out the molecular testing. MW quantified the adenovirus stocks and established TCID_50_ values. TA and KB performed statistical analyses. JL analyzed the DNA sequencing results. RT, SW and KP were involved in the phylogenetic analyses and typing of the adenoviruses as well as preparing the specificity panels. All authors were involved in the preparation of the manuscript. All authors have read and approved the final manuscript.
